# EMPOWERING LGBTQ OLDER ADULTS: PROMOTING HEALTH EQUITY AND WELL-BEING ACROSS THE LIFE COURSE

**DOI:** 10.1093/geroni/igad104.0704

**Published:** 2023-12-21

**Authors:** Hyun-Jun Kim, Karen Fredriksen-Goldsen

## Abstract

The population is becoming more diverse in terms of sexual orientation and gender identity across all age groups. Lesbian, gay, bisexual, transgender, and queer (LGBTQ) older adults are found to experience health disadvantages over time. However, there is a lack of research on health-related mechanisms, so it is important to understand the unique needs and resources in these underserved communities. This symposium fills this research gap by examining the factors that affect LGBTQ health trajectories and subgroup differences using a life course developmental framework, the Health Equity Promotion Model (HEPM). The HEPM model highlights historical, social, environmental, and cultural influence on health and well-being and varying adaptations of LGBTQ older adults. The symposium draws on data from the first national and longitudinal study, Aging with Pride: National Health, Aging and Sexuality/Gender study, which has been following 2,450 LGBTQ midlife and older adults since 2014. Dr. Nelson will discuss sexual and gender identities and their associations with physical and psychological health-related quality of life. By investigating the interplay between marginalization, health behaviors, and health care access over time, Dr. Fredriksen-Goldsen assesses quality of life over time by gender and generational differences. Dr. Kim will discuss the role of social connectivity and intersectionality in predicting cognitive impairment over time among racially and ethnically diverse LGBTQ older adults. Using a health equity lens, this symposium will shed new light on the ways in which historical and environmental contexts influence human development and how marginalization and resilience shape lives over time. This is a Rainbow Research Group Interest Group Sponsored Symposium.

